# Laryngeal and vocal analysis in bulimic patients

**DOI:** 10.1590/S1808-86942010000400011

**Published:** 2015-10-19

**Authors:** Cynthia P. Ferreira, Ana Cristina Côrtes Gama, Marco Aurélio Rocha Santos, Mariana Oliveira Maia

**Affiliations:** a Speech and Hearing Therapist - Hospital da Santa Casa de Misericórdia de Belo Horizonte; b Speech and Hearing Therapist - PhD in Communicaton Disorders - Federal University of São Paulo (UNIFESP); Adjunct Professor - Department of Speech and Hearing Therapy of the Federal University of Minas Gerais (UFMG); c PhD in Sciences - Graduate Program in Otolaryngology - UNIFESP, ENT - UFMG; d ENT - Hospital Felício Rocho. Universidade Federal de Minas Gerais

**Keywords:** bulimia, bulimia nervosa, voice disorders, larynx, voice

## Abstract

Bulimia is an eating disorder classified as a mental disorder according to DSM-IV.

**Aims:**

The aim of the study was to evaluate vocal and laryngeal abnormalities in patients with bulimia compared to a control group.

**Materials & methods:**

Study control group. Twenty-two women were evaluated, with an age range of 18 to 34 years old. Eleven diagnosed with purging bulimia and 11 in the control group. Both groups underwent an otolaryngological, perception and acoustic evaluation. The statistic analysis was done through a chi-square test and a Kruskall-Wallis non-parametric test, considering 5% as significance level.

**Results:**

The bulimic group presented a higher prevalence of laryngeal abnormalities compared to the control group (p=0.000). The group with bulimia had higher GRBSI values (p=0.000) and A (p=0.022) of the GRBASI scale. The results of vocal acoustics analysis of the jitter, shimmer, PPQ and APQ were higher in the bulimic group (p=0.033). No statistical significance difference in the fundamental frequency and NHR were found between both groups.

**Conclusion:**

the bulimic patients in this study presented more laryngeal, acoustics and perception evaluation disorders when compared to a control group.

## INTRODUCTION

Bulimia is a mental disorder usually characterized by the compulsive and fast ingestion of large quantities of food, followed by inadequate measures to avoid weight gain and a morbid fear of gaining weight. According to the DSM-IV diagnosis, bulimia can be broken down into two clinical subtypes: the purgative - in which they use laxatives, diuretic and self-inflicted vomiting in order to make up for the high caloric ingestion. The other type is associated with fasting and excessive physical activities, in order to avoid weight gain^1^.

There are few studies in the world literature discussing the influence of self-inflicted vomiting in patients with bulimia and its association with vocal and laryngeal changes.

A study carried out with three patients who used their voices professionally, diagnosed with bulimia and with vocal complaints, showed the following results pursuant to their ENT exam: mild-to-moderate dysphonia; laryngeal changes (laryngeal micro-diaphragm; subepithelial hemorrhage, mucosal wave reduction, hyperemia, superficial telangiectasia and polypoid degeneration). Based on these findings, the authors decided to carry out a study in order to assess the influence of vomiting as a cause of vocal and laryngeal changes in patients with bulimia. We assessed ten bulimic women, chosen randomly. The methodology used was laryngoscopy and the auditory perception of voice. Clinical findings were: vocal fold subepithelial hemorrhage, acquired hemangioma, telangiectasia, polypoid lesion, vocal fold edema, posterior laryngeal erythema. Speech disorders found immediately after vomiting were: hoarseness and a lower fundamental frequency^2^.

Another study reported three cases of bulimic patients who came for ENT care with vocal and laryngeal symptoms. Clinical history revealed that the patients had the habit of vomiting after meals in order to maintain body weight. The most prevalent otolaryngological findings among these patients were: build up of secretion and salivary stasis in the pyriform sinuses and the presence of a laceration area and small adherence clots on the back of the tongue caused by an object^3^.

A study involving eight bulimic women, from whom four also had been diagnosed with laryngopharyngeal reflux, used a questionnaire of vocal and laryngeal symptoms, auditory perception assessment and laryngeal evaluation. The authors reported that all the patients had vocal complaints such as hoarseness, laryngeal pain, vocal fatigue and hawking. 75% of the individuals had lower vocal pitch and had aphonia episodes, besides a burning sensation that affected 50% of the patients. Otolaryngological findings revealed the prevalence of posterior cricoid edema (100%), laryngeal edema (75%), posterior region hypertrophy (75%), telangiectasia and polypoid lesions (50%)^4^.

A Brazilian study assessed 11 women diagnosed with purgative bulimia by means of speech and hearing and ENT evaluations found that the laryngeal and vocal symptoms more commonly reported were: hawking and the sensation of globus pharyngeus in 90.9% of the subjects. In the auditory perception evaluation by means of the GRBASI scale, the scores which were more frequently found were of mild degree in almost all the parameters. The most prevalent laryngeal findings were the presence of a thick secretion on the larynx of 45.4% of the patients, middle-posterior triangular cleft and mucosal thickening in the interarytenoid region, both happened to 36.3% of the patients^5^.

Among clinical complications from patients with bulimia, we stress: gastrointestinal and oral changes, followed by frequent hydro-electrolytic disorders. Gastric dilatation may happen as a complication of hyperphagia, besides a delay in gastric voiding and bowel motility. Frequent self-inflicted vomiting causes a loss of the nausea reflex and relaxation of the lower esophageal sphincter. The high occurrence of vomits fosters esophagitis. Among oral changes, we stress palate, pharynx and gingival erythema; tongue lesions and erosions to the dental enamel, and the most frequently affected teeth are the canines and the incisives. There is also an increase in the incidence of cavities. Another important change is parotid gland hypertrophy and, more rarely, the submandibular glands. Clinical manifestations have their magnitude shown according to the level, quantity and frequency of the regurgitation^6^.

As to contact duration and the necessary frequency to produce lesion on the laryngeal mucosa, some studies prove that the minimum laryngeal and pharyngeal exposure to gastric secretion can cause changes^6^. Severe inflammatory responses can be developed by intermittent episodes of laryngopharyngeal reflux^12^. According to these authors, six applications of acid and pepsin during two weeks to the larynx can cause ulcerations on the cricoid cartilage^7^.

Only one study with bulimic individuals controlled the duration of exposure to vomit. In this study, the patients self-inflicted vomiting up to 20 times per day^2^. On this aspect, one can question if, as it happens to laryngopharyngeal reflux, the acid arising from the vomit contacts the larynx? As far as bulimia is concerned, the important issue is to know whether the larynx is exposed to gastric content by means of the frequent vomits on the same way, as it happens in laryngopharyngeal reflux, and if such exposure can, in a similar way, damage the larynx, since constant self-inflicted vomiting can damage the larynx, causing vocal and laryngeal consequences. This response becomes complex since constant vomit self-infliction, as previously mentioned, can cause gastric dilatation and relaxation of the lower esophageal sphincter, making it easier for the gastric content to return to the aesophagus^8^.

Currently, many studies correlate vocal and laryngeal signs and symptoms of extraesophageal reflux with the chemical aggression suffered by the adjacent laryngeal mucosa inherent structures^9^, ^10^, ^11^. These studies reveal that the culprits for these lesions are the acid and pepsin present in the gastric secretions. The acid can, through protein denaturation, damage the mucosa under situations in which the acid concentration causes a pH below 4.0. Pepsin is able to digest cell protein in an acid medium, which action causes mucosal damage^12^.

One highly important element in this entire process is to understand the laryngeal mechanisms involved during a vomit episode, having seen that it is an essential component in order to understand the consequences on laryngeal structure and on the patient's voice.

The laryngeal's physiological activity is vital for human beings, since it is part of breathing and also of the protection mechanisms of the lower airways during swallowing, besides activities associated with speech, and, in a secondary way, of laugh, delivery, defecation and micturition^13^. It is believed that during vomiting, the larynx helps protect the lower airways and helps to raise intra-abdominal pressure, together with the intercostal muscles by means of glottal closure with effort. The larynx goes up, the vocal folds close and, following that, the vestibular folds close, the hyoid bone gets closer to the thyroid cartilage, activating the thyroid-median fold. In this case, the vestibular folds would protect the vocal folds from getting in contact with the gastric secretions. Some authors suggest that the vocal process has a complex protection mechanism against gastric secretion infiltration during vomit^14^. According to them, the aryepiglottic fold would form a first stage of closure, followed by the closure of the vestibular folds, preventing the acid regurgitation to reach the vocal folds. The anatomical and physiological differences between vomit and the gastroesophageal reflux have been approached in several studies.

Another author believes that during the retrograde flow (vomit), there is a reflex preventive apnea, which remains during the entire retrograde pressurization. Together with this, in video-fluoroscopic observations, one can notice that the hyoid bone is kept in a rest position or even lowers a little during descent, coming closer to the larynx. The epiglottis suffers a partial eversion, which causes epiglottis tubercle apposition against the vestibular folds. With this, it is assumed that the pre-epiglottic fat pad takes active participation on the pressurization and resistance increase in the airways, being similar to airway protection during swallowing. According to this author, reflux requires special consideration. The retrograde flow can pass through the interarytenoid space or penetrate at the level of the laryngeal vestibule and trigger episodes of defensive apnea (abrupt and intense reflex closure of the glottis). Thus, the secretions easily permeate and contact the laryngeal structures. Laryngeal and pharyngeal receptors, especially those on the high anterior wall of the laryngo-pharynx, trigger the process and one can see forced and repeated hawking-type expiration^15^.

One important piece of information to be analyzed concerning the vocal findings in a study with bulimic patients after the vomit episodes. According to the authors, the findings were: hoarseness and lower fundamental frequency than what is common^2^. With that we have the doubt on the true problems that frequent vomiting can cause to the larynx. To reach a conclusion for these issues is still a very complex task, especially due to the difficulty in understanding the pathophysiological mechanisms involved in self-inflicted vomiting and the real consequences it has on the larynx.

The goal of the present study was to assess laryngeal signs by means of videolaryngoscopy, and the vocal signs in an auditory perception and acoustic way in bulimic patients, comparing them to individuals without the disorder.

## MATERIALS AND METHODS

We had 22 women in this study. Of them, 11 were women without diagnosis of bulimia, from an age range of 19 to 37 years, with mean age of 24.8 years, as the control group. None of these individuals used medication, nor diuretics and/or laxatives frequently. The other group was made up of bulimic patients, aged between 18 and 34 years, with mean age of 23.8 years. The diagnosis time of bulimia varied between 2 and 14 years, with mean age of 5.8 years. The frequence of vomiting episodes per day varied between 1 and 20 times, with an average of 11.7 episodes/day. The medication used by the patients included antidepressants, anxiolythic, benzodiazepines, antipsychotic, antiepileptics, diuretics and laxatives.

This is a cross-sectional, case-control study. The bulimic patients were randomly selected among those being followed up in our ward, by means of analyzing patient chart and talking to the physician in charge of the clinical care. Exclusion criteria for both groups were: smokers and/people who make professional use of their voices; and for the group of bulimic patients those who did not self-inflict vomiting during the study period.

The individuals freely agreed to participate in the study by signing the free and informed consent form. Then, they were submitted to a questionnaire deployed by the same examiner, with the goal of identifying the data concerning age, types of medication used, time of disease and frequency of vomit episodes.

After answering the questionnaire, all the patients were submitted to a speech and hearing evaluation by means of perception auditory and acoustic voice analysis and also ENT evaluation.

In order to collect the vocal data for this study, we asked the patients to comfortably utter the sustained vowels |a| and |é| in modal register as well as chained speech samples, in this study represented by the days of the week and also to count numbers (1 to 10), in ascending order. The voices were recorded directly in a Dell® computer, Optiplex GX260 model, professional Direct Sound® sound board, coupled to a professional unidirectional condenser Shure® 16A microphone, setup on a tripod, inside a sound treated booth in order to avoid capturing room sound. For such registers the individuals were standing up, 10 cm away from the microphone, at a 45° angle in relation to the patient's mouth, so as to avoid picking up breathing noise during recording.

The recordings were done in a sound-treated booth, with noise below 50 dBSPL (Sound Pressure Level), measured through a digital sound pressure level measuring device from Radio Shack® (cat. N° 33-2055).

After recording the sound wave, the |a| vowel sounds were analyzed in the CSL software, MDVP module, from Kay Elemetrics® and the following acoustic analysis options were analyzed: Fundamental frequency(fo) in Hz, jitter in percentage, Frequency Disturbance Ratio (FDR) in percentage, shimmer in dB, Amplitude Disturbance Ratio (ADR) in percentage and Harmonic-noise ratio (HNR) in dB.

The value of the fundamental frequency used was the mean value of all the frequency periods extracted.

The parameters we chose to measure frequency disturbance were jitter expressed in percentage, which is the value of the relative average of the frequency variation in relation to the period, and the FDR expressed in percentage, which is the relative mean value of the frequency disturbance from 5 to 5 periods (five points average).

The parameters to we used measure the amplitude disturbance were: shimmer expressed in percentage, which is the relative mean value of the amplitude variation, peak-to-peak, and the ADR in percentage, which is the relative mean amplitude value at every 11 periods (mean of 11 points).

The noise average utilized was the PHR, which associates the harmonic component with the noise component of the acoustic wave.

The auditory perception assessment was carried out by three speech and hearing therapists - all with more than five years of experience with voice care. These professionals received previous auditory training, with the goal of establishing a consensus on the concepts and levels of the vocal parameters analyzed. The experiment was blind so as to avoid interference from the examiners on the voice analysis and utterances from the two groups studied, and for that they were presented in a random fashion. For this assessment we used the GRBASI scale^16^. The parameters evaluated were: G-grade, R-roughness, B-Breathiness, A-asthenia, S-stress and I-instability. Each one of these parameters was classified in a 0-3 scale, 0 meaning without alteration; 1= mildly changed; 2- moderately changed; and 3= severely altered.

ENT evaluation was carried out by means of videostroboscopy, made up of rigid optic fiber scope from Mashida®, model LY-CS30, 150 Watt xenon light source from Storz®, model 8020, Toshiba® model IK-CU43 video camera, Philips, model VR788 VCR, Sony® monitor, Sony® VHS videocassette tape, model T-120EDE, SP recording speed. The exam was done according to the classic mode, with the person seated and with the open mouth, tongues out and kept out by a finger forceps involved in gauze, after that, the optic fiber is introduced all the way to the hypopharynx and larynx. During the exam, the individuals were instructed to breath normally and utter the “i” and “e” vowels, with loudness and intensity as close as possible to normal speaking, respectively. The videolaryngoscopic assessment parameters were: vocal fold movement and free borders; mucosal color; mucosal wave type; glottal closure, interarytenoid and supraglottic regions.

The statistical methodology used in this study was made up by the chi-square test to compare the laryngeal findings between the two groups and the non-parametric Kruskall-Walis test used to compare the results from the auditory perception and acoustic analysis.

The level of significance adopted was 5%, assigning an asterisk (*) to the statistically significant values.

This study was approved by the Ethics in Research Committee under protocol number ETIC 359/05.

## RESULTS

The laryngeal findings in the videostroboscopy are compiled on Table 1. The most frequent finding in the bulimic group was a thick secretion build up in the larynx, followed by medial-posterior triangular cleft and mucosal thickening in the interarytenoid region. Other alterations were seen in lesser frequency. Of the 11 patients evaluated, 2 (18.2%) did not have changes in the ENT exam with the posterior triangular cleft.

**Table 1:** Laryngeal findings from both groups studied

	Control Group		Bulimic Group	
Laryngeal findings	N	%	N	%
Build up of thick secretion in the larynx	–	–	5	45,4
Medial posterior triangular cleft	–	–	4	36,3
Mucosa thickening in the interarytenoid region	1	9,1	4	36,3
Posterior triangular cleft	3	27,3	2	18,2
Free border thickening of the vocal folds	–	–	1	9,1
Hyperemia on the posterior third of the left vocal fold	1	9,1	–	
Edema on the posterior third of the left vocal fold	1	9,1	–	
Laryngeal signs suggesting pharyngeal GER	1	9,1	–	
Polypoid lesion	–	–	1	9,1
Double spindle cleft	–	–	1	9,1

Legend:

N= number of events

%=percentage

Person's chi-square test = 16.010 P = 0.000*

The most frequent finding seen in the control group was the posterior triangular cleft. The other signs observed were hyperemia and edema on the posterior third of the left vocal fold and suggestive signs of pharyngeal GER, all found in the same individuals (9.1%). Of the 11 participants from the control group, 10 (90.9%) did not have changes in the otolaryngological exam.

Comparing the presence and absence of laryngeal changes between the groups, the bulimic group had a greater occurrence of changes when compared to the control group (Table 1).

The scores from the auditory-perception analysis of the vocal samples are organized on Table 2, according to the parameters assessed. The data related to the bulimia group shows that most of the patients had a mild degree of general voice change (G1), followed by moderated degree (G2) and, only 1 (9.1%) of the patients had neutral voice quality (G0). Roughness was identified, being mild (R1), in 5 (45.4%) of the patients. We can also notice that 5 (45.4%) participants had mild breathiness (B1); 1 (9.1%) had moderate degree of breathiness (B0) in their vocal samples. As far as asthenia goes, only 1 (9.1%) of the subjects had a mild degree (A1). Stress was mild (S1) in 2 (18.2%) of the individuals. The instability component was the parameter that occurred most often among all parameters assessed, being moderate (I2) in 2 (18.2%) participants and mild (I1) in 4 (36.3%).

**Table 2:** Average values concerning degree of severity of the GRBASI scale parameters in the groups studied.

GRBASI scale	Control group	Bulimic group	p Value
G	0,27	1,16	0,000*
R	0,09	0,60	0,000*
B	0,09	0,63	0,000*
A	0,00	0,21	0,022*
S	0,00	0,36	0,000*
I	0,06	0,74	0,000*

Legend:

N= number of event

%=percentage

Kruskall-Wallis test with P<0.05*

The data from the control group showed that 3 (27.3%) individuals had mild voice change (G1); the other volunteers had neutral vocal quality (G0). Roughness was identified in only one (9.1%) of the individuals, being considered mild (R1). We also noticed that one (9.1%) participant had mild breathiness (B1). Within the samples we did not find asthenia or stress. As far as instability goes, only one (9.1%) volunteer presented this parameter, being considered mild (I1).

All the parameters of the GRBASI scale showed medium values on the severity scale which were higher in the group with bulimia (Table 2).

The values of acoustic measures of fundamental frequencies in Hz, jitter in percentage, frequency disturbance ratio (FDR) in percentage, shimmer in percentage, amplitude disturbance ratio (ADR) in percentage and harmonic noise ratio (HNR) in dB are presented on Table 3.

**Table 3:** Mean values of the acoustic parameters in the groups studied.

Acoustic Parameters	Control group	Bulimic group	p Valor
Fo (Hz)	217,80	231.84	0,201
Jitter (%)	20,5	41,5	0,033*
Shimmer (%)	2,93	3,97	0,033*
FDR (%)	0,27	0,57	0,033*
ADR (%)	2,05	2,72	0,033*
HNR (dB)	0,11	0,12	0,670

## DISCUSSION

This study stemmed from one first descriptive paper,^5^ for the need to investigate whether bulimia nervosa would be a risk factor for the development of vocal and laryngeal changes. Study design: case-control. It is known that feeding behavior disorders have a multifactorial etiology and because of its syndromic character it represents a major challenge for specialists to treat^17^. Bulimia is a field of growing interest, not only in the individual clinical playing field, but it is also an issue of public health. This is mainly due to the significant increase in the prevalence of bulimia nervosa in recent years and the high index of comorbidity that this disorder brings about^18^.

The people affected by this disorder have inadequate weight control practices, excessive concern with weight and diet, dissatisfaction and body image distortion, besides a desire to lose weight^8^.

Morbidity and mortality associated with bulimia nervosa are marked^19^. Clinical complications inherent to this disease are many and are primarily associated with the degree of body weight loss and with inadequate compensatory methods to control weight, such as using diuretics, enemas, laxatives and vomit self-infliction^8^.

Since bulimia is a mental disorder, one must consider the drug influence on the speech and voice of these patients. Although this study did not aim at studying the influence of drugs on the voice of bulimic patients, such variable must be considered, because of scientific proof that some medication can cause voice disorders.

The medications used by patients with bulimia include antidepressants, anxiolithics, benzodiazepines, diuretic and laxatives. Two (18.2%) patients reported not having used medication.

Control group patients did not use any medication; therefore, further studies are necessary in order to assess the possible influence of medication in the voice of bulimic patients.

As far as laryngeal findings are concerned, the greatest occurrence happened in the build up of thick laryngeal secretion, found in 5 (45.4%) of the bulimic patients. The same was not found in any of the individuals from the control group (Table 1). This was also a common finding among all the individuals from a study involving 8 bulimic patients^4^ and in two patients from three case studies^3^. Studies with patients with laryngopharyngeal reflux also reported the presence of a thick laryngeal secretion build up^6^. We believe such fact can be closely associated to the degree of dehydration of these patients. It is known that frequent concomitant vomit episodes with other purgative methods, such as the use of laxative and/ or diuretic agents, which can generate hydro-electrolytic changes and, consequently, dehydration^8^. The increase in mucous viscosity can make its clearance difficult and consequent build up of secretion on the laryngeal structures. However, we must also consider the effects of the gastric acid content on the mucociliary movements of the laryngeal epithelium. Some studies correlate the acid reflux with the mechanisms reduction in cell ciliary activity^20^, therefore, the acid action can be a potential factor associated with the build up of secretion in this region.

The medial posterior triangular cleft happened in 4 (36.3%) of the bulimic patients and it was not found in any of the control group individuals. The so called medial posterior triangular cleft can be an indication of a hyperkinetic setting, in other words, an excessive contraction of the intrinsic laryngeal muscles^21^, ^22^. This type of cleft is defined as being typical of dysphonia caused by muscle stress or dysphonia caused by the skeletal muscle stress syndrome^21^. In another study, such finding was associated with laryngopharyngeal reflux or bulimia. It is plausible to assume that these patients may be developing hyperfunction to compensate for the dysfunctions caused by bulimia. The mucosal thickening in the interarytenoid region happened in 4 (36.3%) bulimic patients, but it was not found in the control group. The study with 8 bulimic patients found 6 (75%) patients with posterior region hypertrophy^4^. Some authors define posterior commissure hypertrophy as one of the most frequent finding in patients with reflux^7^.

Other, less frequent, changes were seen among bulimic patients, such as the posterior triangular cleft in 2 (18.2%), and this was the laryngeal sign most often observed in 3 (27.3%) individuals. This is a characteristic which is commonly found among young women, because of the female glottic configuration, which is shorter in the ventral-dorsal direction, without; however, bringing about any negative impact on voice production^23^. It is also important to stress that, in the telelaryngoscopic exam; the incomplete closure degree can be higher, since such position is not physiological because of tongue traction in protrusion^24^.

The thickening of the vocal folds' free border, the polypoid lesion and the double spindle cleft were found in one (9.1%) individual. Polypoid degeneration is the result of chronic irritation, being of chemical or traumatic origin, in which reflux is an important contributing factor^5^^,^^7^. Bulimia associated with dysphonia has been described in the literature as being associated with polypoid lesions, being present in 4 (50%) of the 8 patients investigated^4^. Another study found unilateral polypoid degeneration in 2 (20%) of the 10 patients analyzed^2^. Regarding the double spindle cleft, we did not find any paper correlating it to reflux or bulimia. It is known that this cleft is very frequent in cases with minimum structural change and secondary mass lesion^21^. In our study, we noticed the presence of this change together with polypoid degeneration. The mucosal thickening on the free border was present in the exam of one patient in the case report and in one (10%) of the bulimic patients randomly assessed2. This type of change is also reported in studies with patients diagnosed with LPR^12^. Usually, this type of lesion stems from speech trauma^25^. Some authors advocate that reflux is closely related to manifestations of dysphonia, because of a hyperfunctional adaptation of the larynx and the chemical damage caused by the acid, which consequences are vocal fold lesions, common in functional or organofunctional dysphonia.

Of the 11 bulimic patients evaluated, 2 (18.2%) did not have changes seen in the ENT exam; in the control group, 10 (90.9%) individuals did not have changes. In these regards it is important to report that we consider laryngeal secretion build up as a change. In another study with 10 bulimic patients, two of them also did not have laryngeal changes^2^.

Comparing the presence and absence of laryngeal changes between the groups, the bulimic group had a greater occurrence of changes when compared to the control group, and such difference was statistically significant, leading us to suggest that bulimia is a risk factor for the development of laryngeal changes.

The auditory perception analysis of the vocal samples was organized on Table 2, according to the parameters evaluated. The data regarding the bulimia group showed that 7 (63.6%) of the patients had mild dysphonia (G1), 3 (27.3%) had moderate degree (G2) and only 1 (9.1%) patient had neutral vocal quality (G0). Roughness was mild (R1) in 5 (45.4%) patients. It was also noticed that 5 (45.4%) of the participants had a mild degree of breathiness (B1); 1 (9.1%) had a mild degree of breathiness (B2) and, among the remaining, 5 (45.4%) patients did not have breathiness (B0) in their vocal samples. As far as asthenia is concerned, only 1 (9.1%) of the individuals had mild degree (A1). Mild Stress (S1) was found in 2 (18.2%) individuals. The instability component was the parameter that occurred the most among the individuals assessed, happening in a moderate degree (I2) in 2 (18.2%) participants and mildly (I1) in 4 (36.3%).

Data concerning the control group showed that 3 (27.3%) of the individuals had mild voice change (G1), among the others, 7 (63.6%) of the volunteers had neutral voice quality (G0). Roughness was seen in only 1 (9.1%) participant, and it was considered mild (R1). We also noticed that one (9.1%) participant had a mild degree of breathiness (B1). In the vocal samples analyzed we did not observe asthenia or stress. As far as instability goes, only one (9.1%) volunteer had this parameter and it was mild (I1).

All the GRBASI scale parameters had higher severity values in the bulimia group, with statistically significance, matching reports from the literature which associates bulimia to vocal signs^26^.

Results from the auditory-perception analysis revealed that most of the patients were considered mildly dysphonic by the examiners, and this was the predominant degree of change in all aspects evaluated. These data corroborate a study carried out with three bulimic patients, who came to the voice clinic, complaining of vocal problems. The degree of dysphonia in these patients ranged between mild to moderate^2^. Another study - case-control - aimed at studying the relationship between excessive vomit in bulimic patients and vocal disorders. In this study, the authors noticed that the difference between them was not statistically significant; therefore, according to them, there is no association between bulimia and vocal changes. Nonetheless, they mention one variable that may have caused confusion and impacted the results since only one patient in the bulimic group reported having self-inflicted vomiting during the evaluation period^14^.

Voice analysis quantifies the sound signal and is considered a more objective and complementary type of auditory-perception analysis, helping to quantify data to describe the correlations of voice quality perception decisions^27^. In the literature we did not find papers discussing short term acoustic data in patients with bulimia nervosa, for this reason we chose to correlate the parameters found with studies in patients with esophageal reflux signs and laryngeal symptoms, although there was no clear association between vomit and LPR concerning larynx and voice.

Fundamental frequency (f0) is defined as the number of vibrations per second produced by the vocal folds. This parameter is established by the vocal fold length, vibrating mass and stress. HNR associates the harmonic component versus the noise component of the acoustic wave, being considered one of the best parameters in clinical application to quantify vocal disorders^28^. In another sample there was no statistically significant difference between the bulimic patients and the control group.

These data corroborate studies done with the population of patients with gastroesophageal reflux and laryngopharyngeal symptoms in the pre-treatment phase with anti gastric acid secretory drugs^28^, ^29^, ^30^.

The frequency disturbance measures, such as the jitter, defined as being the cycle-to-cycle fundamental frequency disturbance, the Frequency Disturbance Ratio (FDR) in percentage and the amplitude disturbance measures, shimmer, which is the cycle-to-cycle amplitude variability, the Amplitude Disturbance Ratio (ADR)^28^ in percentage terms were higher in the group with bulimia, and such difference was statistically significant. All of these parameters also vary in studies which use the voice acoustic analysis as assessment tool, the shimmer is the most significant acoustic parameter^29^.

As it has been discussed, this topic bears great investigative projection, both considering an increase in the prevalence of bulimia, as well as for the need for more reliable methodologies.

Future studies using more objective methodologies, such as pH measurement for 24 hours in two channels, which is the gold standard test to correlate laryngeal exposure to the risk factor and the possible findings stemming from it, are necessary so as to help us understand the action of frequent vomiting on the larynx and on voice production.

## CONCLUSION

1) The group with bulimia had a greater occurrence of laryngeal changes when compared to the control group, and such difference was statistically significant.
2) All GRBASI scale parameters had higher severity values in the group with bulimia, and this was statistically significant.
3) The results from fundamental frequency measures and HNR in both groups are similar, without statistical significance. The values of jitter, shimmer, FDR and ADR are higher in the group with bulimia - a statistically significant difference.
